# The Value of Contrast-Enhanced Ultrasound in the Diagnosis of Cesarean Scar Pregnancy

**DOI:** 10.1155/2016/4762785

**Published:** 2016-05-31

**Authors:** Xi Xiong, Ping Yan, Chunyan Gao, Qiulei Sun, Fenglian Xu

**Affiliations:** Xinqiao Hospital, Third Military Medical University, Chongqing 400037, China

## Abstract

*Objective*. To evaluate the value of contrast-enhanced ultrasound (CEUS) in the cesarean scar pregnancy (CSP).* Methods*. Clinical data from 92 patients with lower uterine segment pregnancy, who underwent conventional ultrasound and CEUS examination in the Department of Obstetrics and Gynecology, were collected by Xinqiao Hospital Third Military Medical University from March 2014 to March 2015. The parameters of ultrasound contrast time-intensity curve (TIC), including arrival time, time to peak, time from peak to one half, basic intensity, peak intensity, and wash-in slope, were analyzed.* Results*. Of the 92 cases of patients with pregnancy in the lower uterine segment, 52 cases were CSP, and 40 cases were intrauterine pregnancy. CEUS was significantly better than conventional ultrasound in terms of sensitivity, negative predictive value, Youden index, and diagnostic accuracy (*P* < 0.05). There was no significant difference in specificity and positive predictive value (*P* > 0.05).* Conclusion*. CEUS has a higher accuracy than conventional ultrasound in diagnosis of CSP.

## 1. Introduction

Cesarean scar pregnancy (CSP) is a relatively rare form of ectopic pregnancy that may lead to life-threatening complications such as severe hemorrhaging and uterine rupture if diagnosis and treatment are late [1, 2]. The incidence of the disease has been rare in the past, but in recent years CSP incidence has shown an increasing trend, with the increased number of cesarean section procedures, especially in China [3–6]. Therefore, the timely and accurate diagnosis of CSP has very important clinical significance. Ultrasound examination is an important diagnostic tool in the clinic. The diagnostic criteria for CSP under ultrasonography are as follows: (1) an empty uterine cavity and cervical canal; (2) development of the gestational sac in the anterior portion of the lower uterine segment; and (3) absence of healthy myometrium between the bladder and the gestational sac. But conventional ultrasound examination can only provide part of the two-dimensional image and some blood flow, and it cannot precisely identify the implantation site of the gestational sac and the blood flow status in a real and intuitive matter. So this may lead to misdiagnoses in some cases. It has been reported that conventional ultrasound's diagnostic accuracy rate is 89.0% for CSP patients [7]. Contrast-enhanced ultrasound (CEUS) is widely used in clinical practice, and its advantage in vascular imaging is increasingly obvious. This study aimed to evaluate diagnostic performance of CEUS imaging in detecting CSP.

## 2. Materials and Methods

### 2.1. Patients

In our study, we included 92 women with early pregnancy who underwent ultrasound examination, which revealed gestational sac located in the lower section of the uterus and decided to terminate the pregnancy, in the Department of Obstetrics and Gynecology, Xinqiao Hospital, Third Military Medical University, Chongqing, China, between March 2014 and March 2015. All women had a history of cesarean section (CS) (72 women with one CS, and 20 women with two or more CS). By comprehensive analysis of various indicators such as laboratory tests, auxiliary examinations, and the surgical conditions, the clinical diagnosis of 52 cases of CSP and 40 cases of intrauterine pregnancy were made. Informed consent was obtained from all patients before performing the contrast-enhanced ultrasound. The study was approved by the ethics committee of Xinqiao Hospital.

### 2.2. Conventional Ultrasound and CEUS

Ultrasonography was performed using an ultrasound system IU 22 (Philips Medical Systems, Bothell, WA, USA) with a transducer frequency ranging from 5.0 to 9.0 MHz. Contrast-specific imaging (CSI) modes were used for CEUS in the ultrasound systems at a low mechanical index (<0.2), which enables effective tissue cancellation to generate almost pure microbubble images and avoids destruction of microbubbles in the circulation.

The examinations and evaluation reports were performed by three sonographers, each of whom had more than 5 years of experience of using ultrasonography to perform gynecologic and obstetric examinations.

A second-generation blood pool US contrast agent, SonoVue (Bracco Imaging S.p.A., Milan, Italy), consisting of phospholipid-stabilized shell microbubbles filled with sulfur hexafluoride gas, was used in this study. In each patient, a dose of 2.4 mL of contrast agent was administered through a 20-gauge cannula embedded in the antecubital vein in bolus fashion (within 1-2 s), followed by a flush of 5 mL of 0.9% normal saline.

The parameters of conventional ultrasound are the size, shape and the implantation site of the gestational sac, the thickness of the uterine scar, and blood flow surrounding the gestational sac. A semiquantitative ultrasound grading system developed by Adler was used for grading blood flow: grade 0 (absent), no blood flow is visualized; grade I (minimal), one or two pixels containing blood flow (usually <1 mm in diameter) are visualized; grade II (moderate), a main vessel and/or several small vessels are visualized; grade III (marked), four or more vessels are visualized. Grade 0-I represents a small volume of blood flow, and grade II-III represents a large volume of blood flow [8].

For quantitative analysis of the CEUS time-intensity curve (TIC) parameters, QLAB image processing software (Netherlands Philips Corporation) version 8.1 was used. The procedure was as follows: the recording of CEUS was played back, the image frame of the uterine scar was selected, and the region of interest (ROI) was set. After sampling the ROI, the computer automatically obtained the time-intensity curve (TIC). The TIC analysis period was 0–180 s. The following parameters were analyzed on the TIC: time parameters: arrival time(s); time to peak(s); time from peak to one half(s); intensity parameters: basic intensity (dB, Decibel); peak intensity (dB); wash-in slope/(dB/s).

### 2.3. Data Analysis

The data were analyzed using SPSS version 17.0 (SPSS Inc., Chicago, IL, USA). The diagnostic ultrasound and CEUS data were evaluated by parametric analysis. The relationship of TIC parameter between CSP and intrauterine pregnancy patients was investigated using *t*-tests. The sensitivity, specificity, positive predictive value (PPV), negative predictive value, Youden index, and diagnosis rate of CSP were computed. Data were expressed as means ± standard deviations or percentages. *P* < 0.05 was considered statistically significant.

## 3. Result

### 3.1. Patients

The patients' ages are ranged from 20 to 48, with a median average age of 31. The mean duration of gestation at diagnosis was approximately 7 weeks (range 5–11). The mean serum *β*-human chorionic gonadotropin concentration was 17543.5 mIU/mL (range 1214.3–84537.4 mIU/mL). All patients were followed up for at least 1 month after curettage.

### 3.2. Diagnostic Results

**Figure 1:**
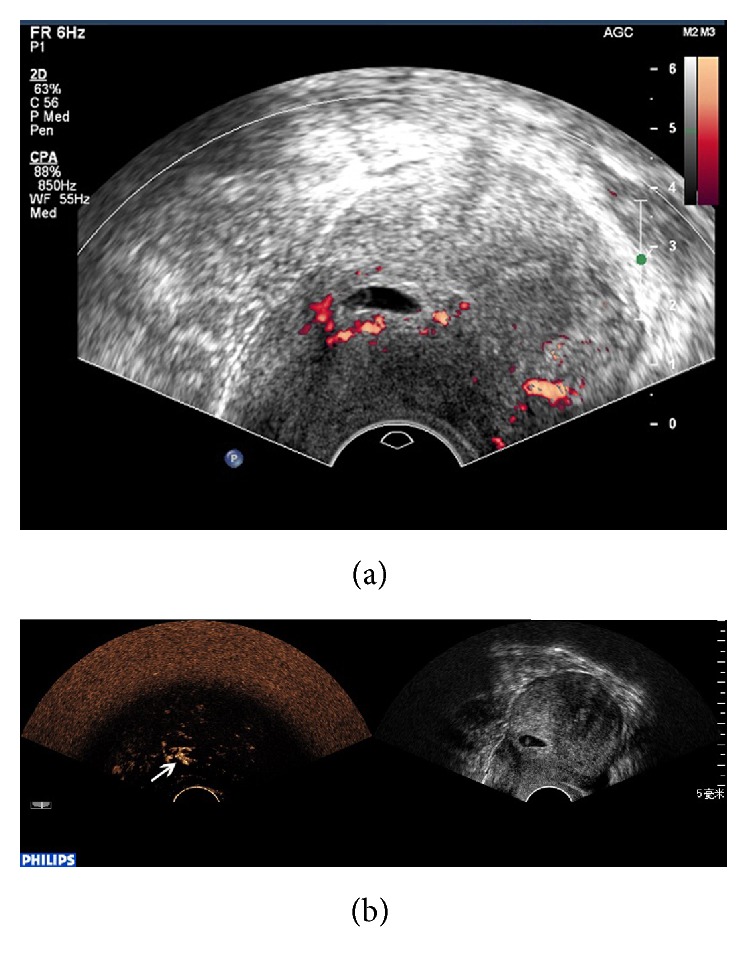


**Figure 2:**
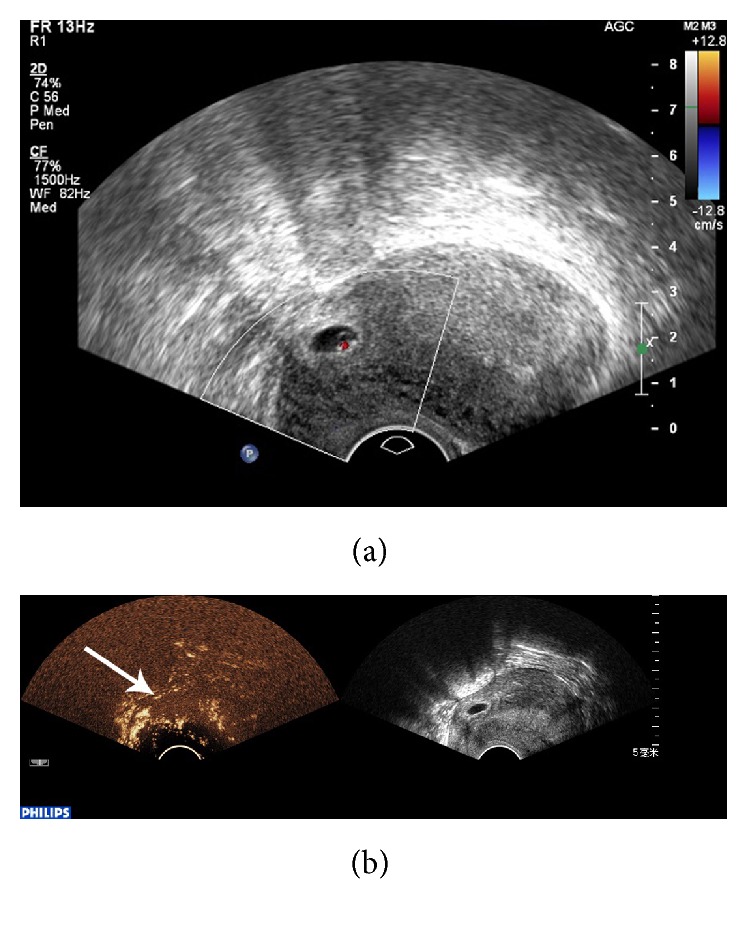


**Figure 3:**
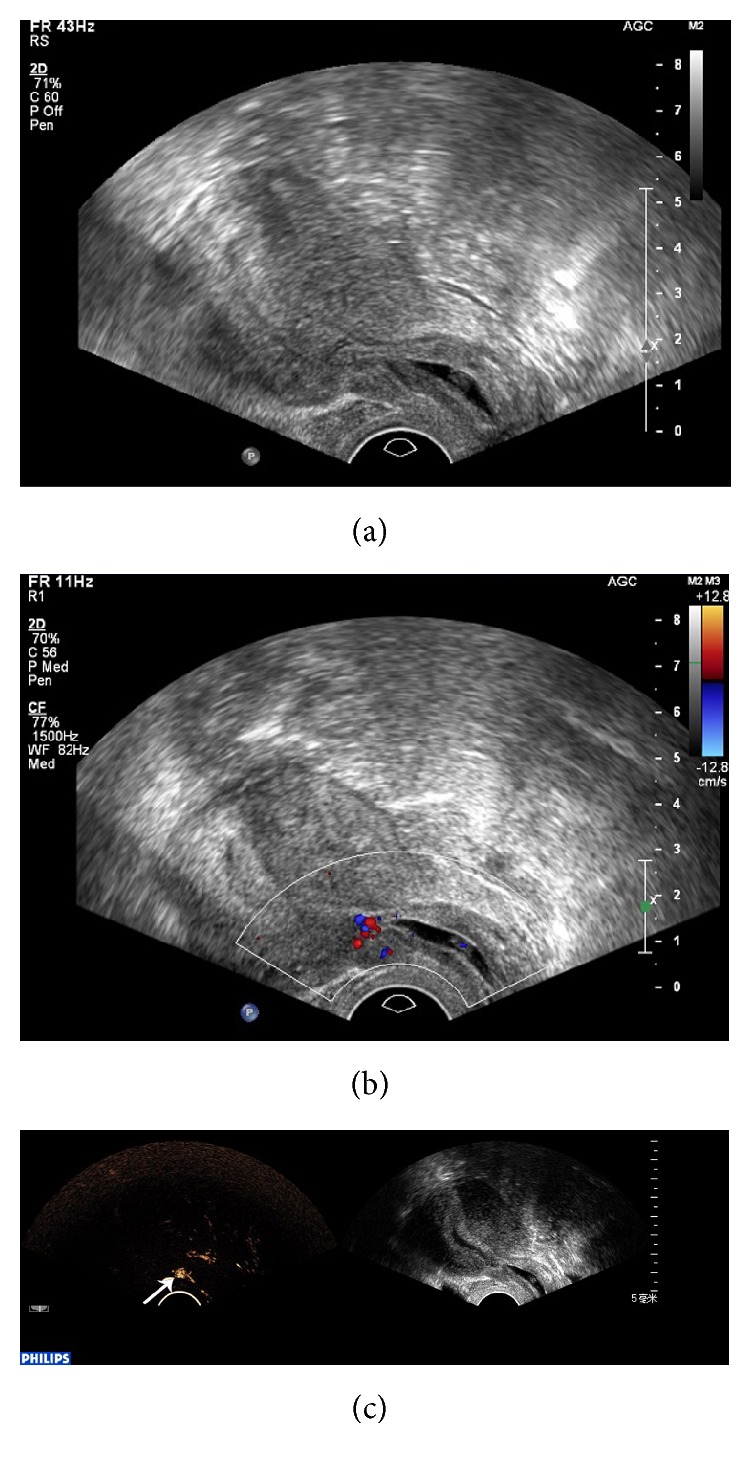


**Figure 4:**
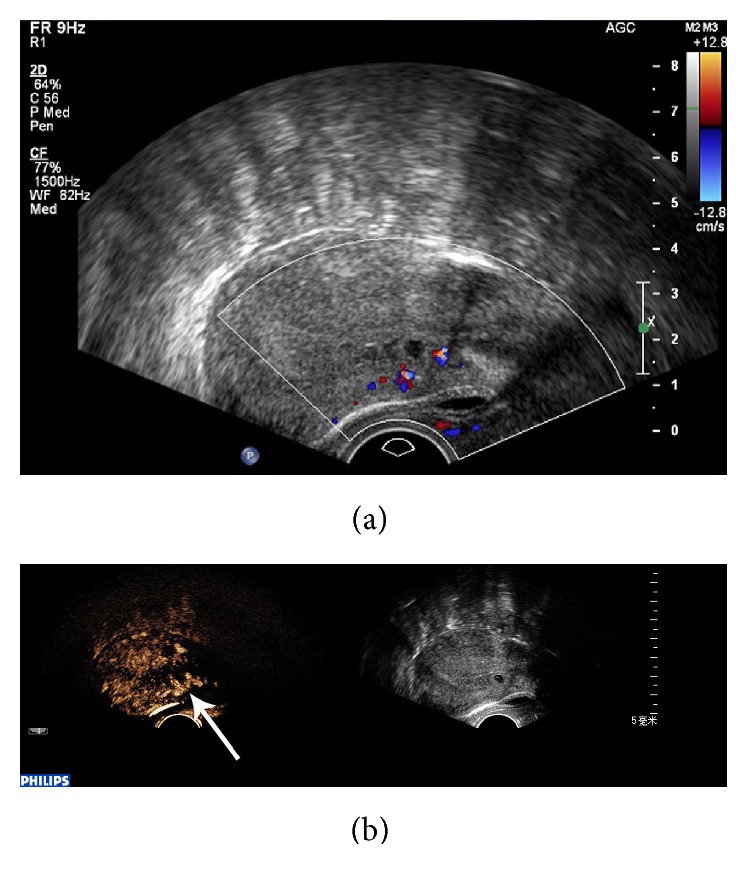


CEUS was significantly better than conventional ultrasound with regard to sensitivity, negative predictive value (NPV), Youden index, and diagnosis rate for CSP (*P* < 0.05), while no significant differences were found with regard to specificity and positive predictive value (PPV) (*P* > 0.05) (Table 1). Conventional ultrasound was accurate in the diagnosis of CSP in 42 cases. 5 cases were misdiagnosed, including 3 cases of intrauterine pregnancy (Figure 1) and 2 cases of inevitable abortion (Figure 2). 10 cases were missed, including 2 cases diagnosed as cervical pregnancy (Figure 3) and 8 cases diagnosed as intrauterine pregnancy (Figure 4). CEUS was accurate in the diagnosis of CSP in 52 cases. Two cases were misdiagnosed as inevitable abortion (Figure 2).

Fifty-two patients with CSP in this study were treated with uterine artery embolization (UAE) following uterine curettage. One patient had an emergency hysterectomy due to postoperative massive hemorrhage, and the others had good recovery without obvious complications with their *β*-HCG decreasing to normal levels within two months.

Forty patients with intrauterine pregnancy were treated with drug abortion or artificial abortion therapy. All the patients recovered well with no obvious complications, and their *β*-HCG decreased to normal levels within one month.

### 3.3. CEUS Imaging

Analysis of the TIC time parameters showed faster arrival time, time to peak, and time from peak to one half (*P* < 0.05) in patients with CSP, when compared with intrauterine pregnancy patients. The TIC intensity parameters showed that CSP patients had a higher peak intensity than that of intrauterine pregnancy patients (*P* < 0.05). There were no significant differences between CSP and intrauterine pregnancy patients in basic intensity and wash-in slope (*P* > 0.05) (Table 2).

## 4. Discussion

Timely and accurate diagnosis of CSP has an extremely important role in clinical practice. Conventional ultrasound has become the preferred imaging method for CSP because it is safe, inexpensive, and simple to perform. But it has limitation in sensitivity to blood flow; there are still avenues for misdiagnosis and missed cases, which reduced its detection rate [3, 7, 9]. CEUS technology develops on the basis of conventional ultrasound, containing tiny bubbles of the principle of acoustic scattering echo enhancement after contrast agent can obviously improve the sensitivity of blood flow, showing the region of interest and the surrounding's perfusion [10–12].

The result showed that both of the conventional ultrasound and CEUS had high specificity and PPV, which means conventional ultrasound is still the first choice to diagnosis CSP. However, when the conventional ultrasound cannot accurately display the location of gestational sac, the CEUS can act as a substitution due to its higher sensitivity, NPV, and Youden index. In clinical practice, we can diagnose the disease more accurately and timely by using CEUS and thus reduce the risk of severe hemorrhaging and uterine rupture greatly.

Because the conventional ultrasound failed to show clearly the source of blood flow to gestation sac, it reduced to 5 cases of misdiagnosis and 10 cases of misdiagnosis. For these types of CSP patients, CEUS can clearly show the blood supply of the pregnancy decidua coming from the uterus scar, indicating the site of embryo implantation. CEUS improves diagnostic accuracy and thus has an edge over conventional ultrasound.

**Figure 5:**
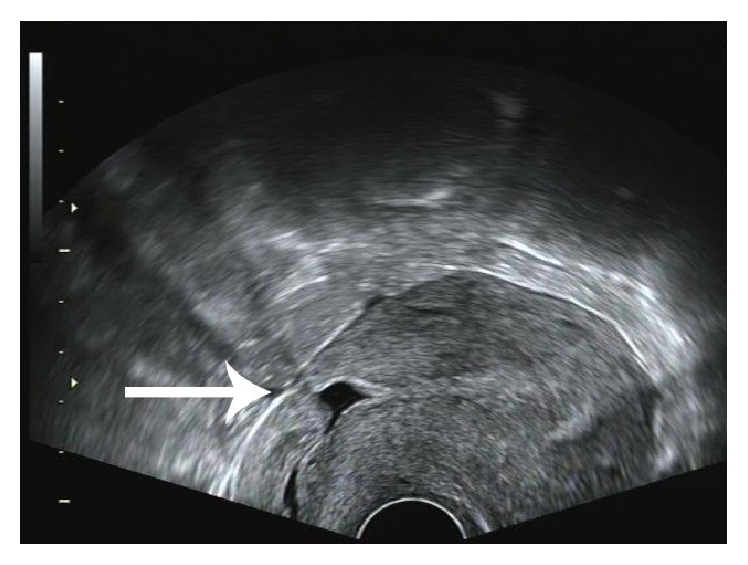


A team discussion was carried out about the two cases in both conventional ultrasound and CEUS misdiagnosed. There were two main reasons: on the one hand, it may be due to poor healing of cesarean section scar and the myometrium in the scar was significantly thin (Figure 5). So when the gestational sac slipped to the defect, the ultrasound can be showed as gestational sac incarcerated in the scar. On the other hand, the ROI did not accurately reflect the position of the pregnancy sac implantation may be due to the two cases had twice cesarean deliveries.

TIC curve is a quantitative calculation method that reflects the microbubbles' volume and flow in the blood vessel as time changes [13]. In this study, we found that CSP patients were significantly different from the control group in arrival time, time to peak, time from peak to one half, and peak intensity. This is consistent with the formation mechanism of CSP that the gestational sac was planted in scars where it can supply the blood of the gestational sac [14].

Since the standard of contrast-enhanced ultrasound diagnosis of CSP had not been reported before, we conducted the study and drew the conclusion as below. As the location of gestational sac in the CSP patients could be fully or partially in uterine scar and also could be in the middle of the uterine cavity and the cervical canal, CEUS can accurately display the location of gestational sac. Another point is that among CSP patients, the parameters of arrival time, time to peak, and time from peak to one half were faster than those in intrauterine pregnancy patients. The TIC intensity parameter displayed CSP patients had a higher peak intensity than intrauterine pregnancy patients.

This study has two limitations. First, some patients in the study had a history of 2 or more cesarean sections, and this could lead to the area of ROI displaying some deviation. Second, the parameter of area under the curve has not been used because some patients with swallowing and slight movements can affect the morphology of the curve (ascending branch and descending branch), and this may result in errors.

Through our comprehensive analysis of the diagnosis results for CSP by means of conventional ultrasound and CEUS, we believe that CEUS can reflect the implantation site of gestational sac more accurately, and the imaging features of CEUS may lead to a more accurate diagnosis before the specific treatment for CSP.

The novelty of this study is that the use of the CEUS in ectopic pregnancies has not been reported before, and the use of the CEUS has an advantage over conventional ultrasound and thus could improve diagnostic accuracy.

## Figures and Tables

**Table 1:** Comparison of two diagnostic methods (%).

	Sensitivity	Specificity	Positive predictive value	Negative predictive value	Youden index	Diagnosis rate
Conventional ultrasound	80.8	87.5	89.4	77.8	68.3	83.7
CEUS	100.0	95.0	96.3	100.0	95.0	97.8
*Z*	3.519	1.198	1.339	3.586	3.216	1.962
*P*	<0.001	0.231	0.181	<0.001	0.001	0.050

CEUS: contrast-enhanced ultrasound; CSP: cesarean scar pregnancy.

CEUS was significantly better than conventional ultrasound with regard to sensitivity, negative predictive value, Youden index, and diagnosis rate for CSP (*P* < 0.05).

**Table 2:** Comparison of TIC parameter between CSP and intrauterine pregnancy patients.

Parameter	Cesarean scar pregnancies (*n* = 52)	Intrauterine pregnancy (*n* = 40)	*t*	*P*
Time parameter				
Arrival time/s	10.91 ± 1.27	17.25 ± 0.89	26.886	<0.001
Time to peak/s	24.73 ± 2.64	39.75 ± 1.83	30.67	<0.001
Time from peak to one half/s	81.56 ± 3.74	79.90 ± 2.97	2.303	0.024
Intensity parameter				
Basic intensity/dB	0.49 ± 0.06	0.50 ± 0.07	0.737	0.463
Peak intensity/dB	19.17 ± 2.04	10.39 ± 1.15	24.384	<0.001
Wash in slope/(dB/s)	1.16 ± 0.16	1.18 ± 0.22	0.505	0.615

TIC: time-intensity curve; CSP: cesarean scar pregnancy; dB: Decibel.

Analysis of the TIC time parameters showed faster arrival time, time to peak, and time from peak to one half (*P* < 0.05) in patients with CSP, when compared with intrauterine pregnancy patients. The TIC intensity parameters showed that CSP patients had a higher peak intensity than intrauterine pregnancy patients (*P* < 0.05). There were no obvious differences between CSP and intrauterine pregnancy patients in basic intensity and wash-in slope (*P* > 0.05).
